# Richmond Medical College, Virginia

**Published:** 1849-08

**Authors:** 


### RICHMOND MEDICAL COLLEGE, VIRGINIA.

Dr. David Tucker, one of the editors of the Medical Examiner, has recently heen appointed Professor of the Practice of Medicine, in the above institution. Dr. Tucker is a gentleman of high professional worth and will doubtless fill the chair to which he has been called with distinguished ability and success.

